# Pregna-1,4,20-trien-3-one, a cytotoxic marine steroid from the marine soft coral *Nephthea* sp.

**DOI:** 10.1107/S1600536810027352

**Published:** 2010-07-17

**Authors:** Maria B. Tabot, Gregor Schnakenburg, Harald Gross

**Affiliations:** a Institute for Pharmaceutical Biology, University of Bonn, Nussallee 6, 53115 Bonn, Germany; b Institute of Inorganic Chemistry, University of Bonn, Gerhard-Domagk-Str. 1, 53121 Bonn, Germany

## Abstract

The title compound, C_21_H_28_O, was isolated from the cytotoxic lipid extract of the Fidjian soft coral *Nephthea* sp. The steroid showed inhibitory activity to human colon adenocarcinoma SW480 cells (IC_50_ = 2.5 µg ml^−1^). The mol­ecular structure indicates that the *A* ring is almost planar (r.m.s. deviation = 0.032 Å), the *B* and *C* rings adopt chair conformations and the five-membered *D* ring is a half-chair. The *B*/*C* and *C*/*D* ring junctions are *trans*-fused.

## Related literature

For chemical background to soft corals, see: Coll (1992 ▶); Sarma *et al.* (2009 ▶). For the initial isolation of the title compound, see: Kingston *et al.* (1977 ▶); Higgs & Faulkner (1977 ▶). For further isolations of the title compound from other organisms, see: Maia *et al.* (1998 ▶); Ciavatta *et al.* (2004 ▶); Zhang *et al.* (2003 ▶, 2005 ▶). Huang *et al.* (2006 ▶, 2009 ▶); Yan *et al.* (2004 ▶, 2007 ▶). For steroid ring conformations, see: Kingston *et al.* (1979 ▶). For further information on the cytotoxicity studies, see: Grever *et al.* (1992 ▶); Ullrich *et al.* (2009 ▶). For a related structure, see: Thompson *et al.* (1999 ▶).
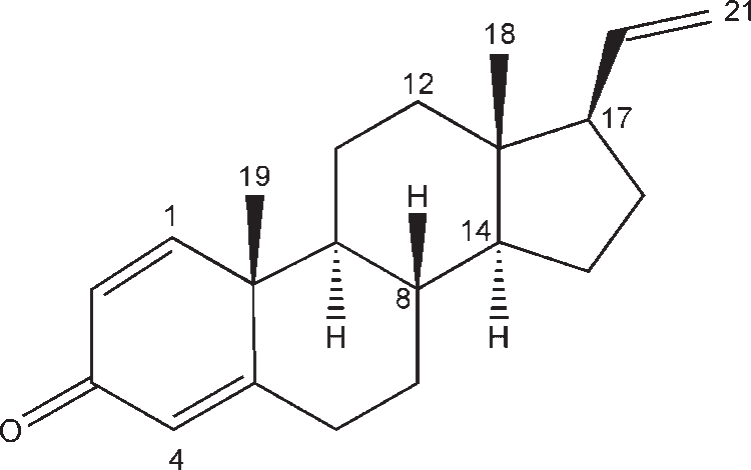


## Experimental

### 

#### Crystal data

C_21_H_28_O
*M* *_r_* = 296.43
Orthorhombic, 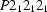

*a* = 6.967 (5) Å
*b* = 11.470 (9) Å
*c* = 20.891 (16) Å
*V* = 1669 (2) Å^3^
*Z* = 4
Mo *K*α radiation
μ = 0.07 mm^−1^
*T* = 100 K
0.32 × 0.16 × 0.06 mm

#### Data collection

Bruker X8 Kappa APEXII diffractometer
7256 measured reflections
2267 independent reflections
1144 reflections with *I* > 2σ(*I*)
*R*_int_ = 0.142

#### Refinement

*R*[*F* ^2^ > 2σ(*F* ^2^)] = 0.063
*wR*(*F* ^2^) = 0.142
*S* = 0.97
2267 reflections
202 parameters
H-atom parameters constrained
Δρ_max_ = 0.25 e Å^−3^
Δρ_min_ = −0.26 e Å^−3^

### 

Data collection: *APEX2* (Bruker, 2009 ▶); cell refinement: *SAINT* (Bruker, 2009 ▶); data reduction: *SAINT*; program(s) used to solve structure: *SHELXS97* (Sheldrick, 2008 ▶); program(s) used to refine structure: *SHELXL97* (Sheldrick, 2008 ▶); molecular graphics: *DIAMOND* (Brandenburg, 1999 ▶); software used to prepare material for publication: *SHELXL97*.

## Supplementary Material

Crystal structure: contains datablocks global, I. DOI: 10.1107/S1600536810027352/hb5516sup1.cif

Structure factors: contains datablocks I. DOI: 10.1107/S1600536810027352/hb5516Isup2.hkl

Additional supplementary materials:  crystallographic information; 3D view; checkCIF report

## Figures and Tables

**Fig. 1.**
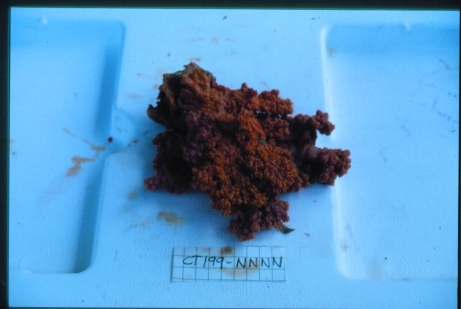
Macroscopic picture of Nephthea sp.

**Fig. 2.**
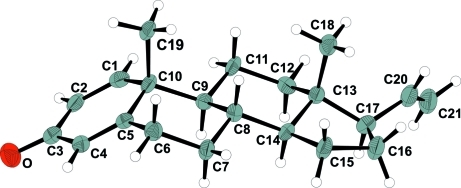
Molecular structure of Pregna-1,4,20-triene-3-one showing 50% probability displacement ellipsoids (DIAMOND; Brandenburg, 1999).

## supplementary crystallographic information

### Comment

Soft corals of the genus *Nephthea* are rich in sesqui- and di-terpenoids (Coll, 1992) and steroids (Sarma *et al.*, 2009). As part of our search for bioactive substances from Fidjian marine invertebrates, the soft coral *Nephthea* sp. (Figure 1) was studied as its CH_2_Cl_2_ extract showed in an initial screening significant cytotoxicity to gastric adenocarcinoma HM02 (IC_50_ = 2.1 µg/ml), hepatocellular carcinoma HepG2 (IC_50_ = 3.2 µg/ml), and breast adenocarcinoma MCF7 (IC_50_ = 4.0 µg/ml) cell lines. Bioassay-guided fractionation resulted in the isolation of pregna-1,4,20-triene-3-one as the major secondary metabolite. X-ray analysis confirmed the structure proposed on the basis of spectral evidence, primarily NMR. Pregna-1,4,20-triene-3-one was first isolated independently in parallel by the groups of Fallis (Kingston *et al.*, 1977) and Faulkner (Higgs & Faulkner, 1977) from the sea raspberry *Gersemia rubiformis* and an unidentified soft coral, respectively. Later on, the steroid was also obtained from soft corals of the genus *Carijoa* (Maia *et al.*, 1998; Ciavatta *et al.*, 2004), *Cladiella* (Zhang *et al.*, 2003; Huang *et al.*, 2006; Huang *et al.*, 2009), *Sinularia* (Zhang *et al.*, 2005) and *Spongodes* (Yan *et al.*, 2004; Yan *et al.*, 2007). However, this is the first time that pregna-1,4,20-triene-3-one has been isolated from a soft coral of the genus *Nephthea*. The molecule contains a fused four-ring system A/B/C/D (Figure 2). Ring A with two double bonds is highly planar (Thompson *et al.*, 1999), whereas the cyclohexane rings B and C adopt chair conformations. The five membered ring D exhibits a half-chair form. The B/C and C/D ring junctions are *trans*-fused. The methyl substituents at atoms C-10 and C-13 are oriented to the same side of the steroid nucleus. The vinyl group at C17 is attached equatorially to ring D. The absolute configuration could not be determined by *x*-ray, but could be established by direct comparison of optical rotation and ^1^H NMR and ^13^C NMR data with literature data (Ciavatta *et al.*, 2004; Higgs & Faulkner, 1977; Kingston *et al.*, 1977 and 1979).

Despite its frequent occurrence and isolation of this metabolite, a biological activity was never attributed to its molecular structure. The cytotoxic effects of purified pregna-1,4,20-triene-3-one against mouse NIH/3 T3 and human SW480 (human colon adenocarcinoma) cell lines was investigated, and the compound showed no activity towards NIH/3 T3 tumor cells, but significant growth inhibitory activity towards SW480 tumor cells (IC_50_ = 2.5 µg/ml). Sterols, particularly highly oxygenated steroids have been reported to be cytotoxic towards several cancer cell lines. However, in contrast to the examples known from the literature, it is noteworthy that pregna-1,4,20-triene-3-one exhibits cytotoxicity with its solely mono-oxygenated structure featuring a rare vinyl group at C-17.

### Experimental

*Animal material*

The soft coral *Nephthea* sp. (Figure 1) was collected in 1999 from Fiji Islands and stored in EtOH at -20°C until workup. A voucher specimen has been deposited at the Institute for Pharmaceutical Biology, University of Bonn, voucher number CT199 NNNN.

*Extraction and isolation*

The freeze-dried soft coral *Nephthea* sp. (250 g dry wt) was extracted repeatedly with CH_2_Cl_2_/MeOH (2:1, *v*/*v*) to produce 10.5 g of crude organic extract. A portion of the extract (5 g) was subjected to normal phase vacuum liquid chromatography (VLC), using stepwise gradient elution from hexanes containing increasing proportions of EtOAc followed by MeOH, to produce nine subfractions. The fraction eluting with 60% EtOAc in hexanes was found to be most active in cancer cell line assays. This fraction was further chromatographed on RP18 solid phase extraction (SPE) cartridges using a stepwise gradient solvent system of decreasing polarity starting from 70% aqueous MeCN to 100% MeCN. The most active fraction after SPE was then purified by normal phase HPLC [Knauer Eurospher-100Si-5 µm (250 *x* 8 mm), petroleum ether / acetone (85:15), 1.5 ml/min, refraction index detection] giving pure pregna-1,4,20-triene-3-one (9.3 mg). Colorless needles of (I) were prepared by slow evaporation from a methanol solution.

*Spectroscopic data*

EI—MS: m/e (*rel*. abundance) 296 (27), 122 (100), 91 (14), 79 (7).

^1^H NMR (300 MHz, CDCl_3_): δ 7.06 (H-1, d, 10.2), 6.21 (H-2, dd, 10.2, 1.7), 6.06 (H-4, t, 1.7), 5.73 (H-20, ddd, 16.7, 10.7, 7.7), 4.98 (H-21*a*, dd, 10.7, 1.5), 4.93 (H-21*b*, dd, 16.7, 1.5), 2.47 (H-6ax, m), 2.36 (H-6eq, m), 1.95 (H-7a, m), 1.95 (H-17, m), 1.78 (H-16*a*, m), 1.72 (12*a*, m), 1.68 (H_2_-11, m), 1.68 (H-15*a*, m), 1.62 (H-8, m), 1.56 (H-16*b*, m), 1.24 (H-15*b*, m), 1.22 (H_3_-19, s), 1.06 (H-9, m), 1.05 (H-12*b*, m), 1.04 (H-7 b, m), 0.99 (H-14, m), 0.65 (H_3_-18, s).

^13^C NMR (75 MHz, CDCl_3_): δ 186.4 (C-3, s), 169.4 (C-5, s), 156.0 (C-1, d), 139.3 (C-20, d), 127.4 (C-2, d), 123.8 (C-4, d), 114.9 (C-21, t), 55.1 (C-17, d), 54.6 (C-14, d), 52.7 (C-9, d), 43.6 (C-10, s and C-13, s), 37.1 (C-12, t), 35.6 (C-8, d), 33.7 (C-7, t), 32.9 (C-6, t), 27.1 (C-16, t), 24.9 (C-15, t), 22.5 (C-11, t), 18.7 (C-19, q), 12.9 (C-18, q).

*Optical rotation*

[α]^20^_D_ +35° (CHCl_3_, c = 0.37)

*Cytotoxicity Assay*

Initial cytotoxic activity of the crude extract towards HM02, HepG2, and MCF7 cancer cell lines were determined by standard procedures (Grever *et al.*, 1992). Human SW-480 (colon adenocarcinoma) and mouse NIH-3 T3 (Swiss mouse embryo) cell lines were obtained from the German Collection of Microorganisms and Cell Cultures (DSMZ) and cultured under conditions recommended by the depositor. The cytotoxicity of pregna-1,4,20-triene-3-one was determined in a viability assay using 3-(4,5-dimethyl-2-thiazolyl)-2,5-diphenyltetrazolium bromide (MTT) as described elsewhere (Ullrich *et al.*, 2009).

### Refinement

All hydrogen atoms were placed in calculated positions with C—H distances ranging from 0.95 to 0.99Å and included in the refinement in riding motion approximation, with *U*_iso_ = 1.2 *U*_eq_ of the carrier atom.

Due to the absence of any significant anomalous scatterers in the compound, the absolute configuration could not be determined from the diffraction data. All Friedel equivalents were merged before the final refinement.

### Crystal data

**Table:**

C_21_H_28_O	*F*(000) = 648
*M**_r_* = 296.43	*D*_x_ = 1.179 Mg m^−^^3^
Orthorhombic, *P*2_1_2_1_2_1_	Mo *K*α radiation, λ = 0.71073 Å
Hall symbol: P 2ac 2ab	Cell parameters from 688 reflections
*a* = 6.967 (5) Å	θ = 2.6–24.9°
*b* = 11.470 (9) Å	µ = 0.07 mm^−^^1^
*c* = 20.891 (16) Å	*T* = 100 K
*V* = 1669 (2) Å^3^	Plate, colourless
*Z* = 4	0.32 × 0.16 × 0.06 mm

### Data collection

**Table:**

Bruker X8 Kappa APEXII diffractometer	1144 reflections with *I* > 2σ(*I*)
Radiation source: fine-focus sealed tube	*R*_int_ = 0.142
graphite	θ_max_ = 28.0°, θ_min_ = 2.6°
fine slicing φ and ω scans	*h* = −8→9
7256 measured reflections	*k* = −10→15
2267 independent reflections	*l* = −24→26

### Refinement

**Table:**

Refinement on *F*^2^	Secondary atom site location: difference Fourier map
Least-squares matrix: full	Hydrogen site location: inferred from neighbouring sites
*R*[*F*^2^ > 2σ(*F*^2^)] = 0.063	H-atom parameters constrained
*wR*(*F*^2^) = 0.142	*w* = 1/[σ^2^(*F*_o_^2^) + (0.0414*P*)^2^] where *P* = (*F*_o_^2^ + 2*F*_c_^2^)/3
*S* = 0.97	(Δ/σ)_max_ < 0.001
2267 reflections	Δρ_max_ = 0.25 e Å^−^^3^
202 parameters	Δρ_min_ = −0.26 e Å^−^^3^
0 restraints	Extinction correction: *SHELXL97* (Sheldrick, 2008), Fc^*^=kFc[1+0.001xFc^2^λ^3^/sin(2θ)]^-1/4^
Primary atom site location: structure-invariant direct methods	Extinction coefficient: 0.009 (2)

### Special details

**Table:**

Experimental. During data collection the crystal was in cold N_2_ gas of a Kryoflex cooler (Bruker AXS).
Geometry. All e.s.d.'s (except the e.s.d. in the dihedral angle between two l.s. planes) are estimated using the full covariance matrix. The cell e.s.d.'s are taken into account individually in the estimation of e.s.d.'s in distances, angles and torsion angles; correlations between e.s.d.'s in cell parameters are only used when they are defined by crystal symmetry. An approximate (isotropic) treatment of cell e.s.d.'s is used for estimating e.s.d.'s involving l.s. planes.
Refinement. Refinement of *F*^2^ against ALL reflections. The weighted *R*-factor *wR* and goodness of fit *S* are based on *F*^2^, conventional *R*-factors *R* are based on *F*, with *F* set to zero for negative *F*^2^. The threshold expression of *F*^2^ > σ(*F*^2^) is used only for calculating *R*-factors(gt) *etc*. and is not relevant to the choice of reflections for refinement. *R*-factors based on *F*^2^ are statistically about twice as large as those based on *F*, and *R*- factors based on ALL data will be even larger.

### Fractional atomic coordinates and isotropic or equivalent isotropic displacement parameters (Å^2^)

**Table:**

	*x*	*y*	*z*	*U*_iso_*/*U*_eq_	
C1	0.0756 (6)	0.8569 (4)	0.1960 (2)	0.0264 (11)	
H1	−0.0301	0.9090	0.1935	0.032*	
C2	0.0531 (6)	0.7591 (4)	0.2299 (2)	0.0279 (12)	
H2	−0.0662	0.7446	0.2505	0.033*	
C3	0.2060 (6)	0.6750 (4)	0.2361 (2)	0.0285 (11)	
C4	0.3822 (6)	0.6981 (4)	0.1998 (2)	0.0255 (11)	
H4	0.4818	0.6415	0.2005	0.031*	
C5	0.4080 (6)	0.7956 (3)	0.1657 (2)	0.0237 (11)	
C6	0.5879 (6)	0.8167 (4)	0.1284 (2)	0.0272 (11)	
H6A	0.6749	0.7490	0.1329	0.033*	
H6B	0.6545	0.8864	0.1454	0.033*	
C7	0.5388 (6)	0.8353 (4)	0.0578 (2)	0.0269 (11)	
H7A	0.6577	0.8528	0.0336	0.032*	
H7B	0.4827	0.7629	0.0400	0.032*	
C8	0.3973 (6)	0.9350 (4)	0.0493 (2)	0.0250 (11)	
H8	0.4591	1.0085	0.0648	0.030*	
C9	0.2129 (6)	0.9137 (4)	0.0890 (2)	0.0233 (11)	
H9	0.1545	0.8404	0.0719	0.028*	
C10	0.2575 (6)	0.8901 (3)	0.1614 (2)	0.0229 (11)	
C11	0.0639 (6)	1.0103 (4)	0.0783 (2)	0.0255 (11)	
H11A	0.1130	1.0840	0.0967	0.031*	
H11B	−0.0557	0.9897	0.1013	0.031*	
C12	0.0181 (6)	1.0290 (3)	0.0069 (2)	0.0254 (11)	
H12A	−0.0463	0.9588	−0.0103	0.031*	
H12B	−0.0710	1.0958	0.0023	0.031*	
C13	0.2001 (6)	1.0527 (3)	−0.0312 (2)	0.0233 (11)	
C14	0.3411 (6)	0.9511 (3)	−0.0204 (2)	0.0247 (11)	
H14	0.2701	0.8788	−0.0328	0.030*	
C15	0.4951 (6)	0.9686 (4)	−0.0717 (2)	0.0310 (12)	
H15A	0.5552	0.8934	−0.0836	0.037*	
H15B	0.5961	1.0227	−0.0567	0.037*	
C16	0.3837 (6)	1.0211 (4)	−0.1290 (2)	0.0336 (12)	
H16A	0.3808	0.9654	−0.1651	0.040*	
H16B	0.4457	1.0941	−0.1436	0.040*	
C17	0.1756 (6)	1.0464 (4)	−0.1046 (2)	0.0265 (11)	
H17	0.0957	0.9762	−0.1143	0.032*	
C18	0.2877 (6)	1.1710 (3)	−0.0131 (2)	0.0294 (11)	
H18A	0.1916	1.2325	−0.0190	0.044*	
H18B	0.3988	1.1868	−0.0405	0.044*	
H18C	0.3286	1.1693	0.0318	0.044*	
C19	0.3332 (6)	1.0012 (4)	0.1957 (2)	0.0280 (11)	
H19A	0.3791	0.9805	0.2386	0.042*	
H19B	0.2294	1.0584	0.1993	0.042*	
H19C	0.4391	1.0347	0.1709	0.042*	
C20	0.0811 (7)	1.1483 (4)	−0.1348 (2)	0.0325 (12)	
H20	0.1523	1.2189	−0.1366	0.039*	
C21	−0.0940 (7)	1.1494 (4)	−0.1594 (2)	0.0394 (13)	
H21A	−0.1702	1.0807	−0.1585	0.047*	
H21B	−0.1435	1.2188	−0.1779	0.047*	
O	0.1903 (4)	0.5870 (3)	0.27010 (17)	0.0409 (9)	

### Atomic displacement parameters (Å^2^)

**Table:**

	*U*^11^	*U*^22^	*U*^33^	*U*^12^	*U*^13^	*U*^23^
C1	0.027 (2)	0.027 (2)	0.025 (3)	0.004 (2)	−0.004 (2)	−0.007 (2)
C2	0.036 (3)	0.031 (2)	0.016 (3)	−0.009 (2)	0.004 (2)	−0.003 (2)
C3	0.037 (3)	0.029 (2)	0.020 (3)	−0.001 (2)	−0.001 (2)	−0.004 (2)
C4	0.031 (2)	0.024 (2)	0.022 (3)	0.005 (2)	0.001 (2)	−0.001 (2)
C5	0.029 (2)	0.023 (2)	0.019 (3)	−0.001 (2)	0.000 (2)	−0.004 (2)
C6	0.027 (2)	0.029 (2)	0.026 (3)	0.003 (2)	−0.001 (2)	0.002 (2)
C7	0.030 (3)	0.032 (2)	0.018 (3)	0.004 (2)	−0.001 (2)	0.004 (2)
C8	0.030 (2)	0.025 (2)	0.020 (3)	−0.001 (2)	−0.001 (2)	0.002 (2)
C9	0.027 (2)	0.021 (2)	0.022 (3)	−0.0005 (19)	0.006 (2)	0.0003 (19)
C10	0.026 (2)	0.029 (2)	0.014 (3)	−0.0060 (19)	−0.001 (2)	−0.0012 (19)
C11	0.030 (2)	0.034 (2)	0.013 (3)	−0.003 (2)	0.004 (2)	−0.001 (2)
C12	0.026 (2)	0.021 (2)	0.029 (3)	0.0010 (19)	−0.001 (2)	0.001 (2)
C13	0.027 (2)	0.024 (2)	0.019 (3)	−0.002 (2)	−0.001 (2)	0.001 (2)
C14	0.032 (3)	0.028 (2)	0.015 (3)	0.0025 (19)	0.003 (2)	−0.002 (2)
C15	0.035 (2)	0.041 (3)	0.017 (3)	0.004 (2)	0.005 (2)	0.004 (2)
C16	0.037 (3)	0.044 (3)	0.020 (3)	0.008 (2)	0.000 (2)	0.005 (2)
C17	0.026 (2)	0.030 (2)	0.023 (3)	−0.001 (2)	−0.001 (2)	−0.004 (2)
C18	0.032 (2)	0.034 (2)	0.023 (3)	−0.003 (2)	−0.007 (2)	0.002 (2)
C19	0.036 (2)	0.032 (2)	0.015 (3)	−0.003 (2)	−0.005 (2)	0.000 (2)
C20	0.038 (3)	0.039 (3)	0.020 (3)	0.002 (2)	0.000 (2)	0.002 (2)
C21	0.046 (3)	0.046 (3)	0.026 (3)	0.009 (3)	−0.003 (3)	0.007 (2)
O	0.047 (2)	0.0356 (18)	0.040 (3)	−0.0040 (17)	0.0076 (19)	0.0089 (18)

### Geometric parameters (Å, °)

**Table:**

C1—C2	1.336 (6)	C12—C13	1.522 (6)
C1—C10	1.508 (6)	C12—H12A	0.9900
C1—H1	0.9500	C12—H12B	0.9900
C2—C3	1.443 (6)	C13—C18	1.535 (5)
C2—H2	0.9500	C13—C14	1.541 (6)
C3—O	1.239 (5)	C13—C17	1.545 (6)
C3—C4	1.466 (6)	C14—C15	1.529 (6)
C4—C5	1.338 (6)	C14—H14	1.0000
C4—H4	0.9500	C15—C16	1.548 (6)
C5—C6	1.495 (6)	C15—H15A	0.9900
C5—C10	1.511 (6)	C15—H15B	0.9900
C6—C7	1.531 (6)	C16—C17	1.564 (6)
C6—H6A	0.9900	C16—H16A	0.9900
C6—H6B	0.9900	C16—H16B	0.9900
C7—C8	1.520 (6)	C17—C20	1.483 (6)
C7—H7A	0.9900	C17—H17	1.0000
C7—H7B	0.9900	C19—H19A	0.9800
C8—C14	1.521 (6)	C19—H19B	0.9800
C8—C9	1.548 (6)	C19—H19C	0.9800
C8—H8	1.0000	C18—H18A	0.9800
C9—C11	1.535 (6)	C18—H18B	0.9800
C9—C10	1.568 (6)	C18—H18C	0.9800
C9—H9	1.0000	C20—C21	1.324 (6)
C10—C19	1.554 (5)	C20—H20	0.9500
C11—C12	1.541 (6)	C21—H21A	0.9500
C11—H11A	0.9900	C21—H21B	0.9500
C11—H11B	0.9900		
			
C2—C1—C10	124.4 (4)	C11—C12—H12A	109.4
C2—C1—H1	117.8	C13—C12—H12B	109.4
C10—C1—H1	117.8	C11—C12—H12B	109.4
C1—C2—C3	121.5 (4)	H12A—C12—H12B	108.0
C1—C2—H2	119.3	C12—C13—C18	111.1 (4)
C3—C2—H2	119.3	C12—C13—C14	108.7 (3)
O—C3—C2	122.0 (4)	C18—C13—C14	112.3 (3)
O—C3—C4	121.2 (4)	C12—C13—C17	114.8 (4)
C2—C3—C4	116.8 (4)	C18—C13—C17	109.3 (4)
C5—C4—C3	122.6 (4)	C14—C13—C17	100.3 (3)
C5—C4—H4	118.7	C8—C14—C15	120.5 (4)
C3—C4—H4	118.7	C8—C14—C13	113.3 (4)
C4—C5—C6	121.7 (4)	C15—C14—C13	104.2 (3)
C4—C5—C10	122.6 (4)	C8—C14—H14	105.9
C6—C5—C10	115.7 (4)	C15—C14—H14	105.9
C5—C6—C7	109.8 (4)	C13—C14—H14	105.9
C5—C6—H6A	109.7	C14—C15—C16	103.9 (3)
C7—C6—H6A	109.7	C14—C15—H15A	111.0
C5—C6—H6B	109.7	C16—C15—H15A	111.0
C7—C6—H6B	109.7	C14—C15—H15B	111.0
H6A—C6—H6B	108.2	C16—C15—H15B	111.0
C8—C7—C6	111.2 (4)	H15A—C15—H15B	109.0
C8—C7—H7A	109.4	C15—C16—C17	106.6 (4)
C6—C7—H7A	109.4	C15—C16—H16A	110.4
C8—C7—H7B	109.4	C17—C16—H16A	110.4
C6—C7—H7B	109.4	C15—C16—H16B	110.4
H7A—C7—H7B	108.0	C17—C16—H16B	110.4
C7—C8—C14	111.7 (4)	H16A—C16—H16B	108.6
C7—C8—C9	110.9 (3)	C20—C17—C13	115.7 (4)
C14—C8—C9	108.6 (3)	C20—C17—C16	114.8 (4)
C7—C8—H8	108.5	C13—C17—C16	103.3 (4)
C14—C8—H8	108.5	C20—C17—H17	107.5
C9—C8—H8	108.5	C13—C17—H17	107.5
C11—C9—C8	111.7 (3)	C16—C17—H17	107.5
C11—C9—C10	113.5 (4)	C10—C19—H19A	109.5
C8—C9—C10	112.3 (3)	C10—C19—H19B	109.5
C11—C9—H9	106.3	H19A—C19—H19B	109.5
C8—C9—H9	106.3	C10—C19—H19C	109.5
C10—C9—H9	106.3	H19A—C19—H19C	109.5
C1—C10—C5	111.9 (3)	H19B—C19—H19C	109.5
C1—C10—C19	105.8 (4)	C13—C18—H18A	109.5
C5—C10—C19	108.9 (4)	C13—C18—H18B	109.5
C1—C10—C9	109.9 (3)	H18A—C18—H18B	109.5
C5—C10—C9	108.6 (4)	C13—C18—H18C	109.5
C19—C10—C9	111.8 (3)	H18A—C18—H18C	109.5
C9—C11—C12	112.4 (4)	H18B—C18—H18C	109.5
C9—C11—H11A	109.1	C21—C20—C17	125.5 (4)
C12—C11—H11A	109.1	C21—C20—H20	117.2
C9—C11—H11B	109.1	C17—C20—H20	117.2
C12—C11—H11B	109.1	C20—C21—H21A	120.0
H11A—C11—H11B	107.9	C20—C21—H21B	120.0
C13—C12—C11	111.0 (3)	H21A—C21—H21B	120.0
C13—C12—H12A	109.4		
			
C10—C1—C2—C3	−0.2 (7)	C8—C9—C10—C19	69.2 (4)
C1—C2—C3—O	176.2 (4)	C8—C9—C11—C12	53.7 (5)
C1—C2—C3—C4	−4.1 (6)	C10—C9—C11—C12	−178.2 (3)
O—C3—C4—C5	−175.9 (4)	C9—C11—C12—C13	−54.9 (4)
C2—C3—C4—C5	4.4 (6)	C11—C12—C13—C18	−68.0 (4)
C3—C4—C5—C6	−179.2 (4)	C11—C12—C13—C14	56.1 (4)
C3—C4—C5—C10	−0.4 (7)	C11—C12—C13—C17	167.4 (3)
C4—C5—C6—C7	121.8 (5)	C7—C8—C14—C15	−54.7 (5)
C10—C5—C6—C7	−57.1 (5)	C9—C8—C14—C15	−177.4 (4)
C5—C6—C7—C8	56.5 (5)	C7—C8—C14—C13	−179.1 (3)
C6—C7—C8—C14	−177.7 (4)	C9—C8—C14—C13	58.3 (4)
C6—C7—C8—C9	−56.4 (5)	C12—C13—C14—C8	−60.0 (4)
C7—C8—C9—C11	−177.0 (4)	C18—C13—C14—C8	63.4 (5)
C14—C8—C9—C11	−53.9 (5)	C17—C13—C14—C8	179.3 (4)
C7—C8—C9—C10	54.2 (5)	C12—C13—C14—C15	167.2 (3)
C14—C8—C9—C10	177.3 (3)	C18—C13—C14—C15	−69.4 (5)
C2—C1—C10—C5	4.0 (6)	C17—C13—C14—C15	46.5 (4)
C2—C1—C10—C19	−114.5 (5)	C8—C14—C15—C16	−161.8 (4)
C2—C1—C10—C9	124.7 (4)	C13—C14—C15—C16	−33.3 (4)
C4—C5—C10—C1	−3.6 (6)	C14—C15—C16—C17	7.2 (5)
C6—C5—C10—C1	175.3 (4)	C12—C13—C17—C20	76.7 (5)
C4—C5—C10—C19	113.0 (5)	C18—C13—C17—C20	−48.9 (5)
C6—C5—C10—C19	−68.1 (5)	C14—C13—C17—C20	−167.1 (3)
C4—C5—C10—C9	−125.0 (5)	C12—C13—C17—C16	−157.0 (3)
C6—C5—C10—C9	53.9 (5)	C18—C13—C17—C16	77.4 (4)
C11—C9—C10—C1	58.4 (4)	C14—C13—C17—C16	−40.8 (4)
C8—C9—C10—C1	−173.7 (3)	C15—C16—C17—C20	148.1 (4)
C11—C9—C10—C5	−178.9 (3)	C15—C16—C17—C13	21.2 (4)
C8—C9—C10—C5	−51.0 (4)	C13—C17—C20—C21	−108.6 (6)
C11—C9—C10—C19	−58.7 (5)	C16—C17—C20—C21	131.2 (5)
